# Machine learning-based prediction of phases in high-entropy alloys: A data article

**DOI:** 10.1016/j.dib.2021.107346

**Published:** 2021-09-16

**Authors:** Ronald Machaka, Glenda T. Motsi, Lerato M. Raganya, Precious M. Radingoana, Silethelwe Chikosha

**Affiliations:** a Advanced Materials Manufacturing, Manufacturing Cluster, Council for Scientific and Industrial Research, P.O. Box 395, Pretoria, 0001, South Africa; b School of Mining, Metallurgy and Chemical Engineering, University of Johannesburg, Johannesburg, South Africa

**Keywords:** High entropy alloys, HEA microstructures, Phases, Machine learning, Deep learning, Material informatics

## Abstract

A systematic framework for choosing the most determinant combination of predictor features and solving the multiclass phase classification problem associated with high-entropy alloy (HEA) was recently proposed [1]. The data associated with that research paper, titled “*Machine learning-based prediction of phases in high-entropy alloys*”, is presented in this data article. This dataset is a systematic documentation and comprehensive survey of experimentally reported HEA microstructures. It contains microstructural phase experimental observations and metallurgy-specific features as introduced and reported in peer-reviewed research articles. The dataset is provided with this article as a supplementary file. Since the dataset was collected from experimental peer-reviewed articles, these data can provide insights into the microstructural characteristics of HEAs, can be used to improve the optimization HEA phases, and have an important role in machine learning, material informatics, as well as in other fields.

## Graphical abstract

**Figure:**
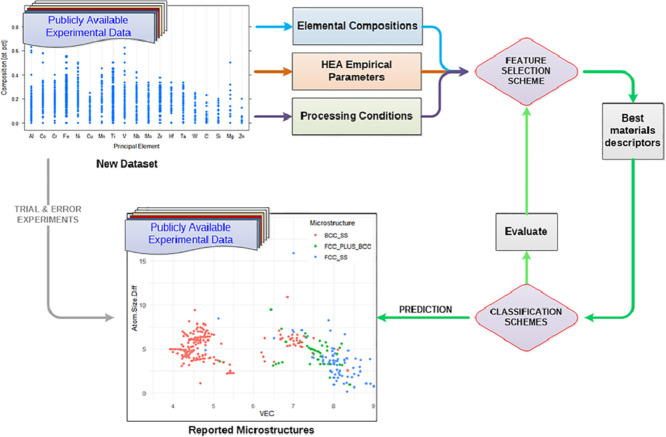

## Specifications Table

**Table:** 

Subject	Materials Science
Specific subject area	High Entropy Alloys (HEA)
Type of data	Text file, tables and figures
How data were acquired	The dataset was gathered from experimental observations reported in peer-reviewed research articles. Each entry of the 1362 dataset entries is therefore an experimental observation as reported in the literature.
Data format	Raw, tabulated and plotted
Parameters for data collection	The following wide range of parameters were collected: i. Identification of the multi-component alloy, ii. the number of elements making up the alloy system, iii. the valence electron concentration, iv. the atomic size difference, v. the difference in Pauling negativities, vi. the enthalpy of mixing for a alloy system, vii. the entropy of mixing of a multi-component alloy system, viii. how the alloy was synthesis and processing conditions (post-process working and heat treatment), ix. the experimentally observed and reported, and x. the bibliographical references against each entry.
Description of data collection	Secondary data (i.e. composition-specific features, alloy processing and post-processing parameters, and the resulting phases) were collected. Some typical empirical HEA design parameters were calculated using well-known approaches. Data was processed using Excel and R, a language and environment for statistical computing, for purposes of visualization and data analysis.
Data source location	Unit: *Advanced Materials Engineering, Manufacturing Cluster* Institution: *Council for Scientific and Industrial Research (CSIR)* Address: *Scientia Campus, 627 Meiring Naude Rd, Brummeria, Pretoria 0185* Country: *South Africa* GPS: *25°44’35.2”S 28°16’52.3”E*
Data accessibility	Data is provided with this article
Related research articles	Research articles associated with research data presented are. [1] Ronald Machaka, *Machine learning-based prediction of phases in high-entropy alloys*, Computational Materials Science, Vol. 188, 2021,11024 https://doi.org/10.1016/j.commatsci.2020.110244

## Value of the Data

• This dataset documents synthesis routes, processing conditions (post-process working and heat treatment), and the resulting microstructural observations which can be valuable for researchers in the field of Materials Science in the development of experiments.
• This dataset specifically contains experimentally reported HEA microstructures which provides enough observations to train and test machine learning and deep learning algorithms.
• Different machine learning and material informatics computational methods can be applied to this dataset inorder to extract insights and trends not immediately available from individual studies thereby advancing the real-world applications of these alloys.

## 1 Data Description

The data presented in this article are related to the research articles [1,2]. The data is presented in the supplementary data file.

### 1.1 The dataset

This dataset is a systematic documentation and comprehensive survey of experimentally reported HEA microstructures. The dataset was constructed from microstructural observations reported in peer-reviewed experimental HEA research articles; it is built upon datasets prior published by Miracle et al. [3], Couzinié et al. [4], and Ye et al. [5]. The dataset presents metallurgy-specific features and microstructural phases experimentally observed.

The dataset, provided with this article as supplementary material, has seventeen columns and 1422 entries.

• Columns 1 and 2 correspond to the identification of the dataset entry and the multi-component alloy system – *Alloy_ID* and *Alloy*, respectively. Composition-specific features can further be developed from the *Alloy* specification
• Columns 3 to 27 correspond to the elemental compositions of the multi-component alloy while Column 28 corresponds to the number of elements making up the multi-component alloy system (*Num_of_Elem*).
• Columns 29 to 37 correspond to some typical empirical HEA design parameters [6,7] such as the density estimate (*Density_calc*), the enthalpy of mixing for a multi-component alloy system (*dH_mix_*), entropy of mixing of a multi-component alloy system (*dS_mix_*), melting temperature estimate (*T_m_*), valence electron concentration (*VEC*), atomic size difference (*δ*), and difference in Pauling negativities (*χ*),. While this article is limited to these typical parameters for succinctness, others can also be developed from the *Alloy* specifications [7], [8], [9], [10], [11], [12], [13].
• Columns 38 to 45 correspond to metallurgy-informed alloy processing and post-processing parameters indicating that: how the alloy was synthesised (*Sythesis_Route*); hot- or cold-worked (*Hot-Cold_Working*); undergone homogenization processing at temperature (*Homogenization_Temp*) and time (*Homogenization_Time*); undergone annealing processing at temperature (*Annealing_Temp*) and time (*Annealing_Time*); and/or undergone some quenching processing (*Quenching*).
• The values tabulated in Column 50 respresent the microstructure or phases corresponding to the multi-component alloy as reported in the literature. Each dataset entry clearly documents the microstructural observations reported for the respective alloy as either single solid solution phases (face-centered cubic – designated *FCC* and body-centered cubic - designated *BCC*), dual-phase solid solutions (designated *FCC+BCC*), other intermetallic, laves, and martensitic phases (designated *Im*).
• The last column, Column 52 provides the bibliographical references against the entries, for transparency.

Table 1 gives a summarised description of these features.

**Table 1:** Descriptions of the empirical and metallurgy-specific features cites ML-based studies that attempted predicting HEA phases therefrom.

Symbol	Description of Feature	References
***Num_of_Elem***	Number of elements in a multi-component alloy system	
***δ***	A parameter describing the atomic size mismatch or difference in a multi-component alloy system $\delta =\sqrt{\sum_{i=1}^{n}c_{i}\cdot {\left(1-\frac{r_{i}}{\bar{r}}\right)}^{2}}$ where $c_{i}$ and $r_{i}$ is the atomic percentage and atomic radius of the *i*^th^ component and $\bar{r}$ is the average atomic radius of the components of the alloy, respectively.	[7], [8], [9], [10], [11], [12], [13]
*Χ*	Pauling negativities mismatch for multi-component alloy system $\chi =\sqrt{\sum_{i=1}^{n}c_{i}\cdot {\left(\chi_{i}-\bar{\chi }\right)}^{2}}$ where $c_{i}$ and $\chi_{i}$ is the atomic percentage and Pauling electronegativity of the *i^t^*^h^ component and $\bar{\chi }$ is the mean value of electronegativity for a multi-component alloy system, respectively.	[7], [8], [9], [10], [11], [12], [13]
***VEC***	The valence electronic concentration of a multi-component alloy system calculated on the basis of the rule of mixtures approach $\mathrm{VEC}=\sum_{i=1}^{n}c_{i}\cdot \mathrm{VE}C_{i}$ where $c_{i}$ and $VEC_{i}$ are the atomic percentage and the valence electron concentration of the *i^t^*^h^ component, respectively.	[7], [8], [9], [10], [11], [12], [13]
Δ***S_mix_***	The entropy of mixing of a multi-component alloy system calculated as follows $\Delta S_{\mathrm{mix}}=-R\cdot \sum_{i=1}^{n}c_{i}\cdot \ln c_{i}$ where ***R*** (= 8.314 JK^−1^mol^−1^) is the universal gas constant and $c_{i}$ is the atomic percentage of the *i*^th^, component.	[8], [9], [10], [11], [12]
Δ***H_mix_***	Enthalpy of mixing for a multi-component alloy system $\Delta H_{\mathrm{mix}}=\sum_{i=1,\;i\neq j}^{n}4\;c_{i}c_{j}\Delta H_{\mathrm{mix}}^{\mathrm{ij}}$ where $c_{i}$ and $c_{j}$ is the atomic percentages of the *i*^th^ and *j*^th^ components, respectively. $\Delta H_{\mathrm{mix}}^{\mathrm{ij}}$ is mixing enthalpy of binary liquid alloys accessible from conventional tables prepared by Takeuchi and Inoue [15] based on Miedema's atomistic model [16].	[8], [9], [10], [11], [12], [13]
***Synthesis_Route***	A categorical feature indicating that the alloy was synthesised via vacuum melted (AC), powder metallurgy (PM), or otherwise	
***Hot-Cold_Working***	A categorical feature indicating that the alloy has been subjected to a cold/hot working treatment. (nan, CW – cold worked, HW – hot worked, HIP – hot-isostatically pressed)	
***Homog_Temp***	A feature indicating the temperature at which the alloy was subjected to a homogenization treatment (in °C)	
***Homog_Time***	A feature indicating the duration for which the alloy was subjected to a homogenization treatment (in minutes).	
***Annealing_Temp***	A feature indicating the temperature at which the alloy was subjected to a annealing treatment (in °C)	
***Annealing_Time***	A feature indicating the duration for which the alloy was subjected to a annealing treatment (in minutes).	
***Quench_Proc***	Categorical feature indicating that the alloy has been subjected to an quenching heat treatment	
***Microstructure***	Experimentally observed microstructure(s), namely *BCC_SS* (body-centered cubic solid solutions), *FCC_SS* (face-centered cubic single solid solutions) *FCC+BCC_SS* (dual-phase solid solutions) and *Im* (intermetallic, intermetallic, laves, martensitic, and other phases but excluding glassy or amorphous and hexagonal close-packed – HCP)	

## 2 Experimental Design and Methods

The dataset is built upon datasets prior published by Miracle et al. [3], Couzinié et al. [4], and Ye et al. [5]. It is constructed from HEA microstructural observations reported in experimental peer-reviewed research articles.

Selected HEAs reported in the literature through the end of August 2020 make up the dataset presented in this article. The as-constructed dataset has at least 1362 multicomponent alloys. After removing entries missing some data and eliminating glassy, amourphous, and hexagonal close-packed phases, the as-constructed dataset is reduced to 1362 multicomponent alloys. Supplementary materials documents accompanying this article contain the full list of alloys and references.

**Fig. 1:**
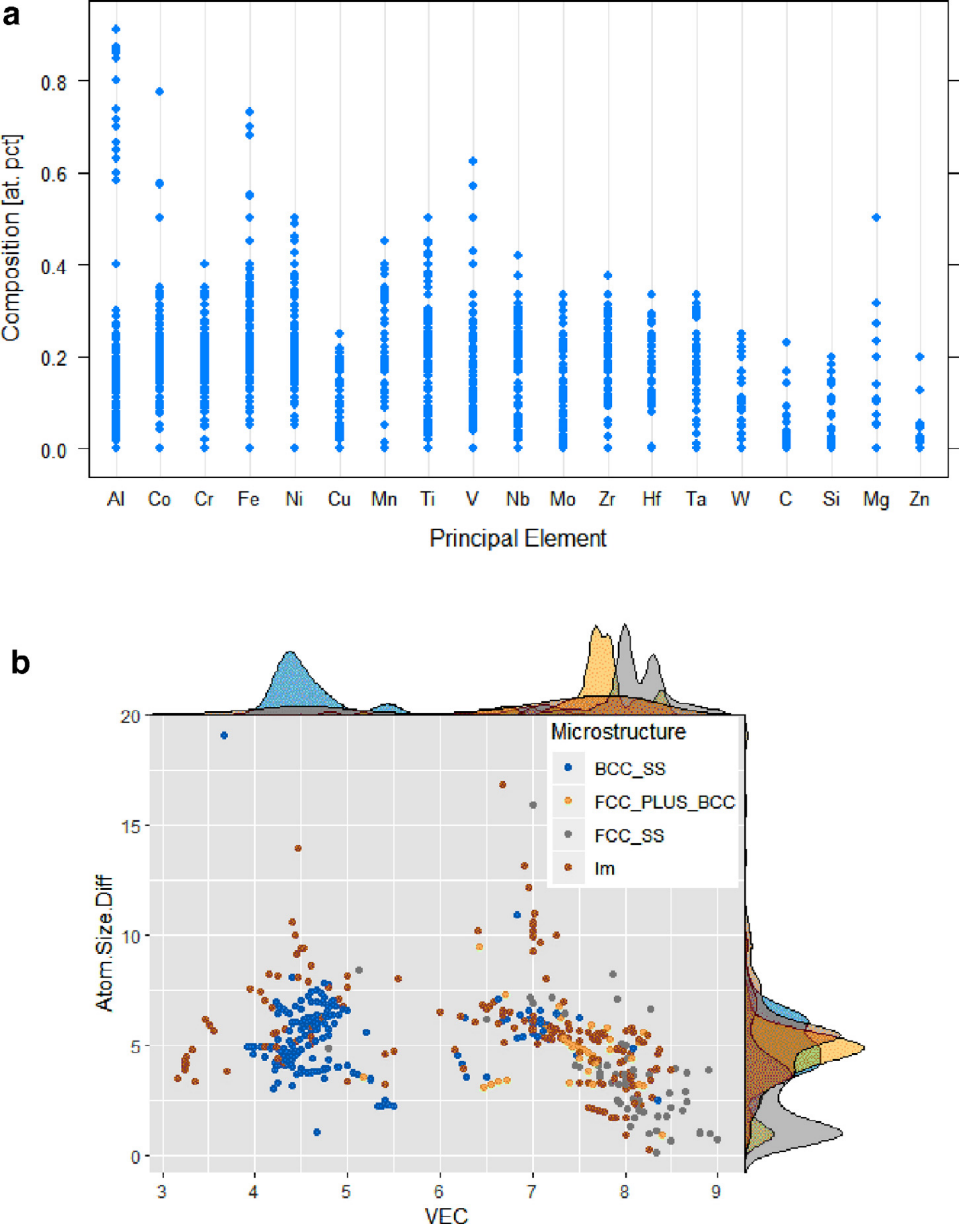
Distribution visualization of the (a) HEA elements and (b) the full dataset phase distribution in the *VEC- δ* space.

The equations used to estimate each of the selected empirical design parameters are summarised in Table 1. Fig. 1 shows the distribution of phase classes in the dataset (the atomic size difference, *δ* as a function of *VEC*) after Refs [7,14]. The visualization also shows that solid solution phases are particularly more sensitive to empirical parameters than IM phases [1,2].

## Ethics Statement

None.

## Data Availability

Dataset for High-Entropy Alloys Phases (Reference data) (Mendeley Data).

## CRediT authorship contribution statement

**Ronald Machaka:** Conceptualization, Funding acquisition, Resources, Methodology, Visualization, Supervision, Writing – original draft, Writing – review & editing. **Glenda T. Motsi:** Data curation, Investigation, Writing – original draft, Writing – review & editing. **Lerato M. Raganya:** Data curation, Investigation, Writing – original draft, Writing – review & editing. **Precious M. Radingoana:** Data curation, Investigation, Writing – original draft, Writing – review & editing. **Silethelwe Chikosha:** Data curation, Supervision, Investigation, Writing – original draft, Writing – review & editing.

## Declaration of Competing Interest

The authors declare that they have no known competing financial interests or personal relationships which have, or could be perceived to have, influenced the work reported in this article.

## Appendix Supplementary materials

Image, application 1
